# High Prevalence of Sinusitis in Children with Henoch-Schönlein Purpura

**DOI:** 10.1155/2011/562638

**Published:** 2011-10-02

**Authors:** Haruna Nakaseko, Osamu Uemura, Takuhito Nagai, Satoshi Yamakawa, Yoshiko Hibi, Yasuhito Yamasaki, Masaki Yamamoto

**Affiliations:** Department of Pediatric Nephrology, Aichi Children's Health and Medical Center, 1-2 Osakada Myoken-Cho, Obu, Aichi 474-8710, Japan

## Abstract

We evaluated the prevalence and the types of infectious foci in oral as well as ear, nose, and throat diseases, and we examined incidence of renal involvement with active treatment for focal infection in children with Henoch-Schönlein Purpura. A total of 96 children who presented at Aichi Children's Health and Medical Center and were diagnosed as having HSP were evaluated for infectious foci in the ear, nose, throat, and oral cavities. Seventy-one of 96 children (74.0%) had some type of infectious lesion, such as sinusitis or tonsillitis, and the prevalence of sinusitis was the highest (51 cases, 53.7%). In 44 HSP patients without renal involvement at the first examination, the incidence of nephritis was lower (13.6%) than in previous reports (17–54%) due to our aggressive intervention for infectious foci.

## 1. Introduction

Henoch-Schönlein purpura (HSP) is the most common type of systemic vasculitis in childhood and is diagnosed when palpable purpura is present plus one of the following: diffuse abdominal pain, any biopsy showing predominant immunoglobulin A (IgA) deposition, arthritis or arthralgia, and renal involvement [1]. Although its cause is unknown, IgA is known to play an important role in its immunopathogenesis. In up to 50% of cases, an upper respiratory infection precedes the disease by days or weeks [2, 3].

Most children with HSP have infectious foci, such as the sinusitis, and therapeutic interventions are often undertaken for these lesions.

In this study, we evaluated the prevalence and the types of infectious foci in oral as well as ear, nose, and throat (ENT) diseases, and the incidence of renal involvement (Henoch-Schönlein purpura nephritis; HSPN) with active treatment for the infectious lesions in children with HSP.

## 2. Materials and Methods

### 2.1. Subjects

A total of 96 children (51 males and 45 females), aged 2–18 years (median: 6 years), who presented at Aichi Children's Health and Medical Center between October 2002 and December 2009 and were diagnosed as having HSP were evaluated for infectious foci in the ear, nose, and throat by an otolaryngologist. Fourteen children in whom we suspected the possibility of endodontic infectious foci based on oral symptoms, such as gum swelling, underwent medical dental examinations.

### 2.2. Infectious Foci

The prevalence and types of these infectious foci were evaluated retrospectively. Forty-four in 96 HSP patients did not have renal involvement at the first examination. We investigated the prevalence of infectious foci in these 44 patients independently and examined whether the incidence of nephritis (HSPN) decreased with provision of sufficient treatment for the infections. 

### 2.3. Bacteriological Examination

We performed nasal cavity culture in 51 cases and throat culture in 14 cases from among the 96 subjects, and conducted an investigation regarding the bacterial types present in the infectious foci.

## 3. Results and Discussion

### 3.1. Results

**Figure 1:**
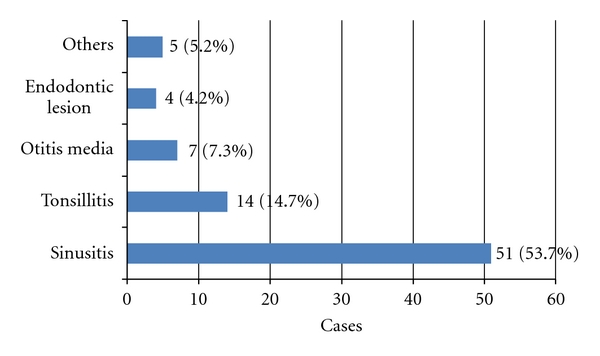
Seventy-one of 96 patients (74.0%) had some type of infections lesion, such as sinusitis or tonsillitis. In these cases, the prevalence of the sinusitis was high (51 cases, 53.7%).

All 96 cases were examined with regard to the presence or absence of focal infection at the otolaryngology department, and 14 of these patients underwent dental examination. Seventy-one of the 96 patients (74.0%) had some type of infectious lesion, such as sinusitis or tonsillitis. Examination of the types of infectious lesions in these 71 cases indicated that the prevalence of sinusitis was the highest (51 cases, 53.7%; Figure 1). 

In 44 patients without renal involvement who could be observed since the onset of HSP, 65.9% had infectious foci. The prevalence of sinusitis among these patients was also high (47.7%). 

The patients with focal infection were treated by administration of antibiotics, sinus irrigation, tooth extraction, and so forth. There was no significant difference in the incidence of renal involvement between the children who did or did not have focal infection (Table 1). Six of the 44 HSP patients (13.6%) developed renal involvement. In addition, one patient who underwent renal biopsy because of persistent proteinuria for two months had minor glomerular abnormalities histopathologically, but none of these six patients required active treatment, such as cocktail therapy.

Table 2 shows the results of nasal cavity culture of 51 patients and throat culture of 14 patients, including overlapping cases. *Haemophilus influenzae *was detected in about 40% of both nasal cavity and throat cultures, and approximately 20% of these were BLNAR (*β*-lactamase-negative ampicillin-resistant *H. influenzae*). *Streptococcus pyogenes* was detected at a low rate in 2.0% of nasal cavity cultures and 7.1% of throat cultures.

### 3.2. Discussion

HSP is a systemic vasculitis disease of the small vessels associated with the deposition of immune complexes containing IgA. The cause remains unknown although there is often an antecedent respiratory infection in half of HSP patients [4]. Widespread abnormalities in IgA have been described, including raised serum IgA concentrations, IgA immune complexes, and IgA class antibodies [5, 6]. In general, HSP is thought to be an IgA immune complex disease, which is caused by mucosal infections.

However, there have been few studies regarding infectious foci in HSP patients. Inoue et al. examined 40 HSP patients for infectious foci, and focal infection was present at a high rate [7]. In thier report, approximately 70% of HSP patients had endodontic infectious foci, such as decayed teeth and periapical periodontitis, and half of these patients showed otolaryngeal infectious lesions, such as sinusitis or otitis media. The incidence of renal involvement and the rate of recurrence of HSP were decreased following interventions for these focal lesions.

Most children with HSP have infectious foci, such as the sinusitis, and often undergo therapeutic intervention for these lesions. We did not regard decayed teeth as focal infections, and therefore the ratio of endodontic focal infection was lower than reported by Inoue [7].

In the present study, a high prevalence of infectious foci was revealed in children with HSP (74%), and the prevalence of sinusitis in these cases was remarkably high (53%).

School medical examinations have been conducted in Japan for more than 30 years, and the prevalence of sinusitis in childhood is regarded as 2.5–3%. Children generally have upper respiratory infection 6–8 times a year, and 5–10% of these cause secondary sinusitis [8]. As the prevalence rate of sinusitis in these HSP patients was markedly higher than in these previous reports, HSP patients with infectious foci appear to have a high risk of renal involvement, protraction, and exacerbation of HSPN. Chronic sinusitis shows spontaneous cure at a high frequency at the age of approximately 7 years [9]. In addition, most cases develop HSP between 2 and 8 years of age; this age distribution was similar for the patients in our study with a median age at onset of 6 years. Considering this evidence, diagnosis and intervention for focal infection, especially sinusitis, should be performed in the treatment of children with HSP and HSPN. Due to aggressive intervention for infectious foci, the incidence rate of renal involvement in 44 HSP patients in the present study treated from onset was lower (13.6%) than that reported previously (17–54%) [10], and none of our six cases with renal involvement required active treatment, such as cocktail therapy. Aggressive intervention for the infectious foci was effective for the treatment of patients with HSP and HSPN.

Sinusitis often cannot be diagnosed in children based only on symptoms, such as postnasal drip or mild productive cough, and we should perform diagnosis and intervention for focal infection, especially sinusitis, in the treatment of HSP and HSPN children even if they do not show obvious symptoms.

*H. influenzae *was detected in about 40% of nasal cavity cultures and throat cultures, and approximately 20% of these isolates were BLNAR. The type of antibiotics administered should be determined in consideration of the antibiotic susceptibility of the causative bacteria.

The results of the present study indicated a high prevalence of infectious foci, especially sinusitis, in children with HSP. We should prospectively examine the efficacy of aggressive intervention for infectious foci in HSP patients.

## 4. Conclusion

A high prevalence of infectious foci was revealed in children with HSP, with an especially high prevalence of sinusitis (53%). We should perform diagnosis and intervention for focal infection in the treatment of HSP and HSPN children to prevent aggravation of the disease.

## Figures and Tables

**Table 1:** Prevalence of renal involvement in total of 44 cases of HSP patients without nephritis at first examination.

	Focal infection (+)	Focal infection (−)
Renal involvement (+)	2	4
Renal involvement (−)	23	15

**Table:** (a) Nasal cavity culture (51 cases)

Haemophilus influenzae (BLNAR*)	19 (37.3%) (5 (9.8%))
Corynebacterium	7 (13.7%)
Moraxella catarrhalis	4 (7.8%)
MSSA**	3 (5.9%)
MRSA***	2 (3.9%)
Streptococcus pneumoniae	2 (3.9%)
S. pyogenes	1 (2.0%)
Others	13 (25.5%)

**Table:** (b) Throat culture (14 cases)

Haemophilus influenzae (BLNAR*)	6 (42.8%) (1 (7.1%))
H. parainfluenzae	2 (14.3%)
S.pyogenes	1 (7.1%)
MSSA**	1 (7.1%)
Others	4 (28.6%)

*β-lactamase negative ampicillin-resistant Haemophilus influenzae.

**methicillin-sensitive Staphylococcus aureus.

***methicillin-resistant Staphylococcus aureus.
